# Genomics reveals multiple introductions of the seventh pandemic Vibrio cholerae O1 El Tor lineage into Iran since 1965

**DOI:** 10.1099/mgen.0.001551

**Published:** 2025-10-30

**Authors:** Bita Bakhshi, Elisabeth Njamkepo, Mozhgan Derakhshan-Sefidi, Mohammad R. Pourshafie, Claire Jenkins, Caroline Rouard, François-Xavier Weill

**Affiliations:** 1 Department of Bacteriology, Faculty of Medical Sciences, Tarbiat Modares University, Tehran, Iran; 2 Unité des Bactéries pathogènes entériques, Centre National de Référence Vibrions et choléra, Institut Pasteur, Université Paris Cité, Paris, France; 3 Department of Microbiology, Pasteur Institute of Iran, Tehran, Iran; 4 Gastrointestinal Bacteria Reference Unit (GBRU), UK Health Security Agency, London, UK

**Keywords:** cholera, Iran, microbial genomics, *Vibrio cholerae* O1, whole-genome sequencing

## Abstract

Cholera epidemics due to *Vibrio cholerae* O1 El Tor have been occurring in Iran since 1965, but the incidence of this disease has declined significantly over time. We found that multiple sublineages of the seventh pandemic *V. cholerae* O1 El Tor lineage have caused cholera outbreaks in Iran, suggesting recurrent introductions of the disease from South Asia into Iran.

**Impact Statement**

*Vibrio cholerae* O1 El Tor, the causal agent of the seventh pandemic of cholera, remains a significant cause of illness and death in South Asia. Iran has experienced regular cholera outbreaks since 1965, but genomic information about this pathogen is scarce in this country. We analysed whole-genome sequences from 74 Iranian *V. cholerae* O1 El Tor isolates collected between 1965 and 2023. The genetic diversity, phylogenetic relationships and temporal distribution of the bacterial genomes studied suggest that the cholera agent has not established a permanent presence in the Iranian environment. Instead, the pathogen is regularly introduced into the country from neighbouring countries, particularly those to the east in which cholera is endemic. This work contributes to global efforts to improve our understanding of the evolution, acquisition of drug resistance and transmission of the causal agent of the seventh cholera pandemic.

## Data Summary

The short-read sequence data generated in this study have been submitted to the European Nucleotide Archive (ENA, http://www.ebi.ac.uk/ena), and their individual accession numbers are listed in Table S1 (available in the online Supplementary Material). All the accession numbers of the publicly available sequences – downloaded from GenBank or ENA – used in this study are listed in Table S2.

## Introduction

**Fig. 1.**
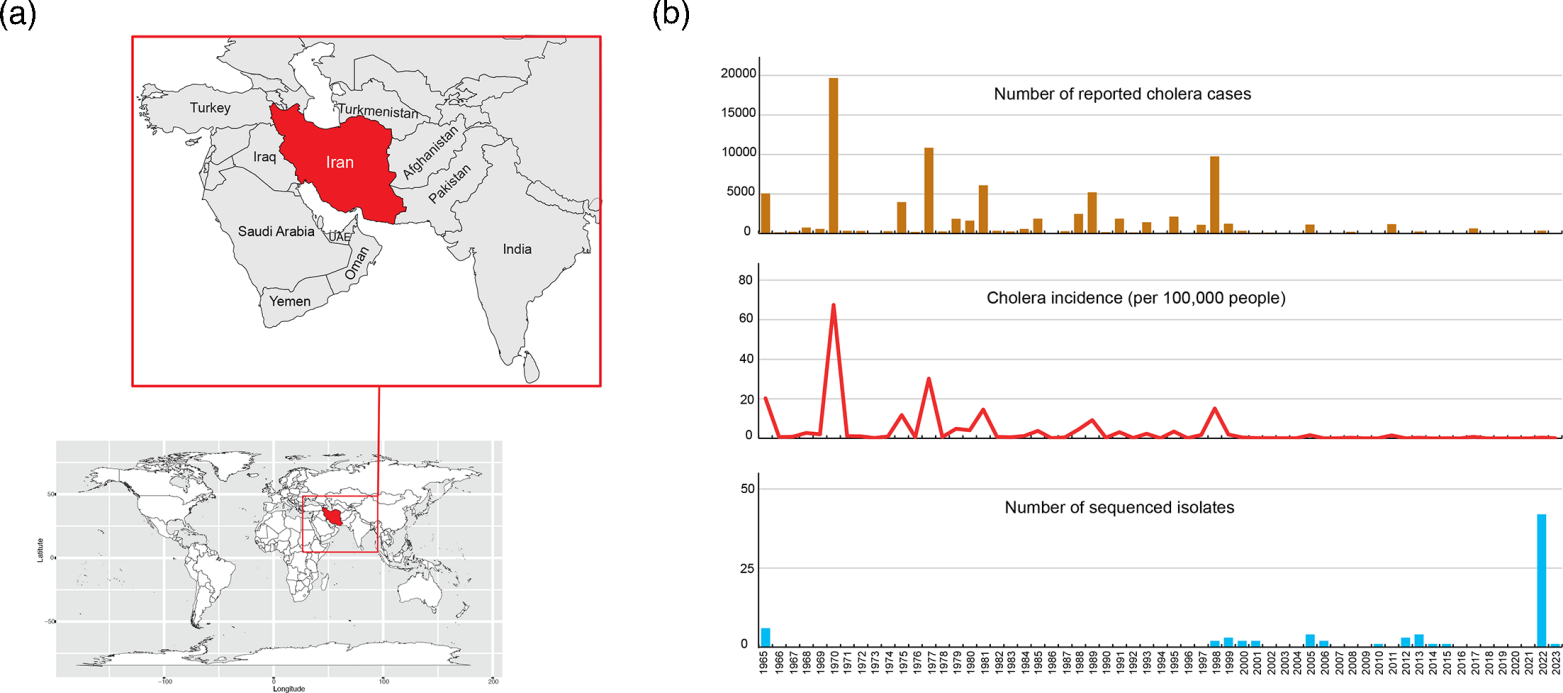
Location of Iran and cholera data for this country over the 1965–2023 period. (a) Location of Iran (in red) on a world map and magnification of the region within the red square showing the neighbouring countries (the names of the main countries are indicated; UAE, United Arab Emirates). Please note that African countries are not shown in the magnification. (b) Number of cholera cases reported, incidence of cholera and number of sequenced *V. cholerae* O1 isolates per year in Iran. The figures used for this chart can be found in Table S3.

Cholera, caused by *Vibrio cholerae* O1 (and more rarely O139), is characterized by severe diarrhoea and fluid loss due to the production of cholera toxin (CTX). About 4.3 million cases and 142,000 deaths occur globally each year [1]. The seventh pandemic El Tor lineage (7PET) is responsible for the ongoing pandemic, which originated in Indonesia in 1961 and has since spread to many areas of the world [2]. Iran, located in southwest Asia and covering 1,648,195 km², had a population of about 90,608,707 people in 2023 (https://data.who.int/countries/364). It shares borders with 13 sovereign countries (including Iraq to the west and Afghanistan and Pakistan to the east) (Fig. 1a). During the seventh pandemic, cholera was first reported in Iran during the summer of 1965, when cases were reported in the eastern part of the country, probably introduced from neighbouring Afghanistan [3]. The disease did not spread across the country but, in total, ~5,000 cases were reported to the World Health Organization (WHO) [3]. Despite two large outbreaks in 1970 (almost 20,000 cases) and 1998 (almost 10,000 cases), the incidence of cholera – which became an urgent notifiable disease at least four decades ago – has greatly decreased over time in Iran, falling to only 79 cases in 2023 according to the National Cholera Surveillance System Database (Fig. 1b) [4]. This decrease in incidence was attributed to better household access to safe drinking water (increase in coverage from 70% to 98% of households) and sanitation (increase in coverage from 66% to 77%), particularly in rural areas, from 1990 onwards [4]. The screening of travellers arriving by land or sea from neighbouring countries in which cholera outbreaks are documented (based on WHO reports) may also have improved control of the disease in Iran [4]. According to the available data, outbreaks occurring before 2001 were attributed to the Ogawa serotype [4]. More recently, there has been an alternation between the Inaba and Ogawa serotypes. Numerous studies based on conventional and molecular typing methods have been performed on *V. cholerae* O1 isolates collected in Iran during the seventh cholera pandemic [3,5,13]. However, only a few studies have been published since the advent of whole-genome sequencing (WGS) for studies of bacterial pathogens, corresponding to fewer than a dozen genomic sequences from Iranian *V. cholerae* O1 isolates [14,16].

We used WGS to study 63 clinical isolates of *V. cholerae* O1 originating from Iran between 1998 and 2023. We also performed a phylogenomic analysis of over 1,600 global 7PET *V. cholerae* genomes, including 11 previously published Iranian genomes (from isolates collected between 1965 and 2022), to determine the genomic diversity and evolutionary history of the cholera agent in Iran during the seventh pandemic.

## Methods

### Bacterial culture, identification and serotyping

We studied 62 *V*. *cholerae* isolates from cholera cases occurring in the Islamic Republic of Iran between 1998 and 2023 [provided by the Pasteur Institute of Tehran (PIT)] and 1 isolate from a cholera case in a traveller returning from Iran in 2023 [provided by the UK Health Security Agency (UKHSA)] in this study (Table S1). At PIT, conventional biochemical tests and/or the API 20 E identification system (bioMérieux, https://www.biomerieux.com) were used to check for the presence of *V. cholerae* in stool cultures [17]. The identity of the isolates was confirmed by PCR targeting an intergenic spacer region specific to the *V. cholerae* species [18]. The serogrouping of isolates was performed with polyclonal O1 and monospecific Ogawa and Inaba antisera (Mast Diagnostics Ltd., Bootle, Merseyside, UK). At UKHSA, the characterization of *V. cholerae* isolates was performed as described previously [19].

### Whole-genome sequencing

Genomic DNA was extracted with the QIAamp DNA Mini kit (Qiagen GmbH, Hilden, Germany), the FavorPrep Tissue Genomic DNA extraction mini kit (Favorgen, Taiwan) or the QIAsymphony system (Qiagen, Hilden, Germany) (UKHSA), in accordance with the manufacturers’ recommendations. The libraries were prepared with the Novogene NGS DNA Library Prep Set (Novogene, Cambridge, UK) or the Nextera XT kit (Illumina, San Diego, USA). Sequencing was performed with the following Illumina platforms: NovaSeq 6000 – at Novogene (Cambridge, UK) or *Institut du Cerveau* (Paris, France) – or HiSeq 2500 (UKHSA), generating 70–150 bp paired-end reads, yielding a mean of 328-fold (minimum 65-fold and maximum 600-fold) coverage. Short-read sequence data were submitted to the European Nucleotide Archive (ENA) (http://www.ebi.ac.uk/ena) and the accession numbers are listed in Table S1.

### Additional genomic data

As a means of placing these 63 7PET isolates into a global phylogenetic context, raw sequence files and assembled genomes from a well-curated representative dataset of 1,554 strains – including 11 published strains originating from Iran and collected in 1965 (*n*=6), 2012 (*n*=1), 2013 (*n*=2), 2015 (*n*=1) and 2022 (*n*=1) [14,16] – were downloaded from the European Nucleotide Archive (ENA, https://www.ebi.ac.uk/ena/) or GenBank (https://www.ncbi.nlm.nih.gov/genbank/). See Table S2 for a list of all 1617 genomes used in this study, with their accession numbers and associated references.

### Genomic sequence analyses

All reads were filtered with FqCleanER v.23.12 (https://gitlab.pasteur.fr/GIPhy/fqCleanER) to eliminate adaptor sequences and discard low-quality reads with phred scores <28 and a length <70 bp, to generate 70–159 bp paired-end reads. Taxonomic read classification with Kraken v.2.1.1 was used to remove contaminated genomic sequences [20]. Only genomes for which >99.5% of reads were assigned to the genus *Vibrio* were retained for further analyses. Short reads were assembled with SPAdes v.3.15.5, with the default settings [21]. Sequence type was determined with the multilocus sequence type scheme of Octavia *et al*. [22]. The various genetic markers were analysed with the Basic Local Alignment Search Tool (blast) v.2.2.26 against reference sequences of the O1 *rfb* gene cluster (*V. cholerae* O1 N16961; GenBank accession no. AE003852; coordinates: 258103–258294), *ctxB* (*V. cholerae* O1 N16961; GenBank accession no. AE003852; coordinates: 1566967–1567341), *wbeT* (*V. cholerae* O1 VX44945; GenBank accession no. JF284685; coordinates: 1–953) and the whole locus of VSP-II (*V. cholerae* O1 N16961; GenBank accession no. AE003852; coordinates: 523156–550021), as previously described [16]. The presence and type of acquired antibiotic resistance genes (ARGs) or ARG-containing structures were determined with ResFinder v.4.3.3 (https://cge.food.dtu.dk/services/ResFinder/), blast v.2.2.26 analysis against GI-15 (*V. cholerae* O1 V060002; GenBank accession no. AP018677; coordinates: 844–29014), Tn*7* (*Shigella sonnei* Ss046; GenBank accession no. CP000038; coordinates: 4098721–4113720) and SXT/R391 integrative and conjugative elements (ICEs): ICE*Vch*Ind5 (*V. cholerae* O1 Ind5; GenBank accession no. GQ463142; coordinates: 1–97952), ICE*Vch*Ban9 (*V. cholerae* O1 MJ-1236; GenBank accession no. CP001485; coordinates: 2959802–3065926), ICE*Vch*Ind4 (*V. cholerae* O139 Ind4; GenBank accession no. GQ463141; coordinates: 1–95326) and PlasmidFinder v.2.1.1 (https://cge.food.dtu.dk/services/PlasmidFinder/). We checked manually for the presence of mutations in the genes encoding resistance to quinolones (*gyrA* [*V. cholerae* O1 N16961; GenBank accession no. AE003852; coordinates: 1330207–1332891] and *parC* [*V. cholerae* O1 N16961; GenBank accession no. AE003852; coordinates: 2603309–2605594]), resistance to nitrofurans (VC0715/*nfsA* [*V. cholerae* O1 N16961; GenBank accession no. AE003852; coordinates: 768551–769273] and VCA0637/*nfsB* [*V. cholerae* O1 N16961; GenBank accession no. AE003852; coordinates: 571824–572477]), or restoring susceptibility to polymyxin B (VC1320/*vprA* [*V. cholerae* O1 N16961; GenBank accession no. AE003852; coordinates: 1404377–1403673]) in sequences assembled *de novo* with blast v.2.2.26 (with default parameters) [15,16].

### Phylogenetic analysis

The paired-end reads and draft or assembled genomes were mapped onto the reference genome of *V. cholerae* O1 El Tor N16961, also known as A19 (GenBank accession numbers LT907989 and LT907990) with Snippy v.4.6.0/BWA v.0.7.17 (https://github.com/tseemann/snippy). Single-nucleotide variants (SNVs) were called with Snippy v.4.6.0/Freebayes v.1.3.2 (https://github.com/tseemann/snippy), under the following constraints: mapping quality of 60, minimum base quality of 13, minimum read coverage of 4 and 75% read concordance at the locus for a variant to be reported. An alignment of core-genome SNVs was produced in Snippy for phylogenetic inference.

Repetitive (insertion sequences and the TLC-RS1-CTX region) and recombinogenic (VSP-II) regions in the alignment were masked [16]. Putative recombinogenic regions were detected and masked with Gubbins v.3.2.0 [23]. A maximum likelihood (ML) phylogenetic tree was built with RAxML v.8.2.12, under the General Time Reversible model with 200 bootstraps [24]. This global tree was rooted on the A6 genome [16] – the earliest and most ancestral 7PET isolate, collected in Indonesia in 1957 – and visualised with iTOL v.6.9.1 (https://itol.embl.de) [25].

## Results and discussion

**Fig. 2.**
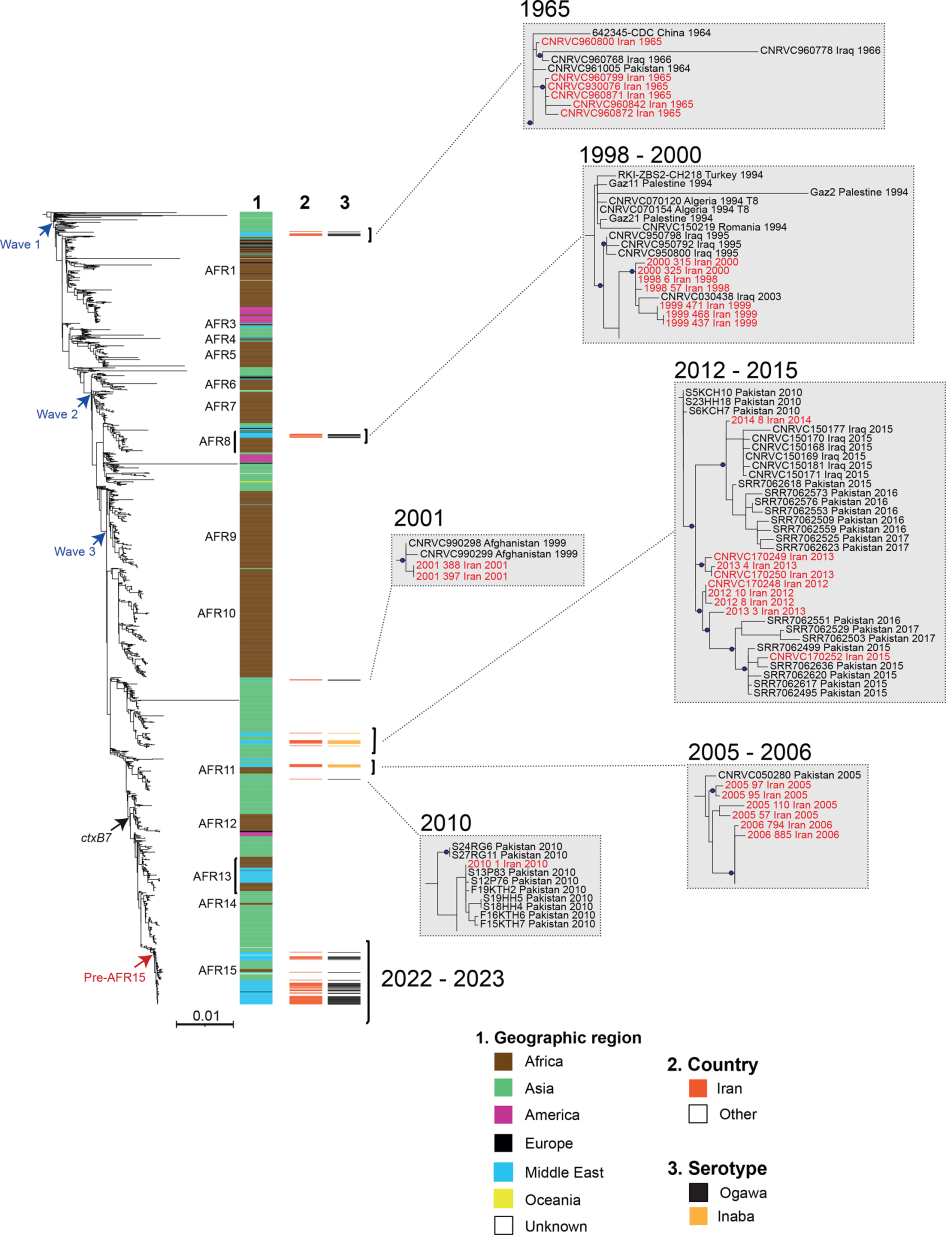
Phylogenomic analysis of 74 *V. cholerae* O1 El Tor isolates originating from Iran collected between 1965 and 2023. This ML phylogenetic tree comprises 1,617 genomic sequences from the seventh pandemic *V. cholerae* biotype El Tor isolates (Table S2). The figure illustrates three genomic waves (in blue) and the acquisition of the *ctxB7* allele (black arrow), with colour coding to indicate the geographic origin of the isolates. Previously described sublineages introduced into Africa (AFR1, AFR3–AFR15) are indicated on the right of the tree. Column 1 lists the geographic origins, whereas column 2 lists 74 isolates from the 1965 to 2023 cholera outbreak in Iran, and column 3 shows the serotype (Ogawa vs. Inaba) of these 74 isolates. Magnifications of the branches of the phylogenetic tree containing isolates from Iran between 1965 and 2015 are shown on the right with red text indicating the Iranian *V. cholerae* O1 isolates studied. For each genome, its name (or accession number), the country in which contamination occurred and the year of sample collection are indicated at the tip of the branch. Blue dots indicate bootstrap values ≥85% for selected nodes. Due to its size, the magnification of the pre-AFR15 sublineage (Iranian isolates from 2022 to 2023) is shown separately as Fig. S1. None of the sequenced isolates were collected between 1966 and 1997. The scale bar indicates the number of nucleotide substitutions per variable site.

We used WGS to characterize 63 *V*. *cholerae* O1 isolates, including 62 collected from cholera cases in Iran between 1998 and 2023 and 1 isolate from a traveller returning to the UK from Iran in 2023 (Table S1). We placed these 63 *V*. *cholerae* O1 isolates in a global context with a collection of 1,554 7PET genomic sequences (Table S2) from around the world – including the 11 previously published Iranian genomes collected from 1965 to 2022 [14,16] – and constructed an ML phylogeny of 1,617 genomes based on 11,061 SNVs evenly distributed over the non-repetitive, non-recombinant core genome (Fig. 2).

Our phylogenetic analysis showed that all 74 isolates (11 previously published isolates and the 63 from this study) from Iran belonged to the 7PET lineage and clustered, according to their year of isolation, within the 3 genomic waves previously described in this lineage [2]. Only the isolates collected in 1965 and grouped in the ancestral part of the tree belonged to wave 1, and only the 1998–2000 isolates belonged to wave 2; all other isolates belonged to wave 3, with the most recent isolates (2022–2023) in the most distal part of the tree (after the acquisition, by the 7PET lineage, of the *ctxB7* variant of the CTX subunit B gene in Southern Asia in the early 2000s [14,16]) (Fig. 2). Regardless of their date of isolation, all Iranian genomes clustered close to genomes from neighbouring countries, such as Pakistan/Afghanistan in the east and Iraq in the west. Most of the 74 isolates belonged to previously described sublineages: the isolates collected in 1998–2000 (*n*=7) belonged to the EUR6/AFR8 sublineage [26], those (*n*=9) from 2012 to 2015 to the AME (Asia/Middle East) sublineage [15] and those from 2022 to 2023 (*n*=43) to the pre-AFR15 sublineage [14,27]. These three sublineages have been shown to circulate in the Middle East, and two of these sublineages (EUR6/AFR8 and pre-AFR15) were eventually introduced into Southern and Eastern Africa.

All recent isolates (2022–2023) from Iran belonged to the pre-AFR15 sublineage. This sublineage was also detected in several Asian countries, including countries neighbouring Iran (Afghanistan with 281,485 reported cases, Pakistan with 1,006 cases and Iraq with 3,708 cases) and other Middle Eastern countries (Lebanon with 5,715 cases and possibly Syria with 70,222 cases, as the first reported case in Lebanon in 2022 occurred in a Syrian refugee, 1 month after the declaration of the Syrian cholera outbreak on 10 September 2022), before it reached Eastern or Southern Africa [14,27,29]. The pre-AFR15 sublineage therefore played an important role in the global resurgence of cholera in 2022. However, the burden of cholera caused by this pre-AFR15 strain was lower in Iran, with only 439 cases reported in 2022–2023 (Table S3), although we cannot rule out a higher burden linked to an underascertainment of cases of mild or asymptomatic cholera and an underreporting of confirmed cases.

All but two of the isolates were serotyped with Ogawa and Inaba antisera (Table S1). The vast majority of the isolates belonged to the Ogawa serotype, and only those collected between 2005 and 2006 or between 2012 and 2015 were of serotype Inaba. A perfect correlation was observed between phenotypic serotype and the sequence of the *wbeT* gene, which encodes a methyltransferase characteristic of the Ogawa serotype. All serotype Ogawa isolates had a WT *wbeT* gene, whereas the serotype Inaba isolates displayed two different alterations to this gene: a non-synonymous SNV (T472) leading to the S158P aa substitution (alteration C13 according to a previous work [16]) in the 2005–2006 Inaba isolates, or an interruption of this gene between nucleotides 400 and 401 by an insertion sequence (IS*Spu7*) in the 2012–2015 Inaba isolates (Table S1).

Antibiotics – particularly tetracyclines – have been used for decades as an adjuvant treatment of cholera in addition to rehydration therapy, as they shorten the duration of diarrhoea, thereby limiting bacterial spread [30]. Antimicrobial resistance (AMR) is an important evolutionary trait in the 7PET lineage and is often linked to the acquisition of various mobile genetic elements, including IncC plasmids and the ICE of the SXT/R391 family, both encoding resistance to multiple antimicrobial drugs [2,16, 31, 32]. The acquisition of SXT/R391 ICEs has been dated to between 1978 and 1984 in the 7PET lineage [2]. This genomic element has since become widespread, leading to subsequent recombination events generating several variants (such as the prevalent ICE*Vch*Ind5 variant, thought to have emerged in about 1985 [33]). The earliest Iranian genomes displaying AMR determinants studied here were collected in 1998–2000 (genomic wave 2 isolates). No pan-susceptible isolates (such as those isolated in 1965) have since been obtained. These 1998–2000 isolates already displayed multiple-drug resistance including resistance to nitrofurans [due to point mutations in the chromosomal genes VC0715/*nfsA* (R169C) and VCA0637/*nfsB* (Q5Stop)], tetracyclines [presence of an acquired gene *tet(A*)], cotrimoxazole (*sul1*, *sul2* and *dfrA1* genes), chloramphenicol (*floR*) and streptomycin (*strAB* and *aadA1*). The acquired AMR genes were located on two genomic elements: GI-15 (*sul1* and *aadA1*) and ICE*Vch*Ban9 [*strAB*, *sul2*, *dfrA1* and *tet(A*)]. However, we cannot rule out the possibility that AMR emerged earlier, as we were unable to locate or resuscitate isolates collected in this country between 1966 and 1997. All the wave 3 isolates had mutations in both the nitrofuran resistance genes and the quinolone-resistance determining region of *gyrA* (S83I), and they subsequently acquired another mutation in *parC* (S85L). Two different SXT/R391 ICEs were identified in the wave 3 Iranian isolates, with the exception of two isolates collected in 2014 (2014/8) and 2015 (2015/8). ICE*Vch*Ind6 [containing *strAB*, *sul2*, *dfrA1* and *tet(59*)] was detected in the 2012–2013 isolates from the Sistan and Baluchestan provinces, and ICE*Vch*Ind5 (containing *strAB*, *sul2*, *dfrA1* and *floR*) was found in all the other isolates. One isolate from 2006 (2006/885) and all 42 from 2022 had a deletion of about 10 kb in ICE*Vch*Ind5, resulting in the loss of the AMR genes *strAB*, *sul2* and *floR*. However, the 2023 isolate (SRR26422249) isolated from a traveller returning to the UK had a complete ICE*Vch*Ind5. No multidrug-resistant IncC plasmids were identified in our dataset. Finally, all the 2022–2023 isolates had a point mutation in the *vprA* gene (VC_1320) resulting in the D89N substitution, which restores susceptibility to polymyxins [15]. Local Iranian databases indicate that there was widespread community access to cheap antimicrobial agents such as beta-lactam antibiotics and then to cotrimoxazole, macrolides (erythromycin), tetracyclines (tetracycline) and fluoroquinolone antibiotics for diarrhoeal and other infections in the late 1990s and early 2000s [34]. These databases indicate that the use of macrolides (azithromycin), tetracyclines (doxycycline) and fluoroquinolone antibiotics increased towards 2016 [34]. Tetracyclines are the first-choice antibiotics for cholera treatment, followed by fluoroquinolones and macrolides [30]. Only the 1998–2000 and 2012–2013 isolates from Iran displayed resistance to tetracyclines. Decreased susceptibility or even resistance to fluoroquinolones has been reported for all isolates tested since 2005. However, none of the isolates from Iran studied here displayed resistance to azithromycin.

In conclusion, this genomic study shows that the multiple cholera outbreaks that occurred in Iran from 1965 to 2023 were caused by different populations of the seventh pandemic *V. cholerae* O1 El Tor. These populations belonged to waves of different 7PET sublineages circulating in Iran for limited periods and were temporally and geographically related to populations in neighbouring countries. The discrepancy between the numbers of cases of cholera reported in Iran and in neighbouring countries therefore probably reflects differences in surveillance intensity, healthcare access and public health interventions rather than differences in pathogen virulence. Our data suggest that there is no permanent reservoir of the cholera agent in Iran and that outbreaks have been fuelled by human migration along routes of cholera spread between South Asia and the Middle East. This hypothesis is consistent with the epidemiological data showing that Iranian border provinces, such as Sistan and Baluchestan, experience recurrent importations of cholera driven by population movements, seasonal trade and border closures [4]. At the national level, cholera cases imported from outside Iranian borders are overrepresented in the years between epidemics [4]. In 2013, for example, 211 (82.4%) of the 256 confirmed cases of cholera in Iran were imported from Afghanistan, which borders Iran to the northeast [4]. The data presented here provide valuable insight into the epidemiology of cholera in Iran, improving our understanding of the global circulation of the 7PET lineage.

## Supplementary material

10.1099/mgen.0.001551 Uncited Supplementary Material 1.

10.1099/mgen.0.001551 Uncited Supplementary Material 2.

## Abbreviations

AMR: antimicrobial resistance
ARG: antibiotic resistance gene
blast: Basic Local Alignment Search Tool
CTX: cholera toxin
ICE: integrative and conjugative element
ML: maximum likelihood
PIT: Pasteur Institute of Tehran
SNV: single-nucleotide variant
UKHSA: UK Health Security Agency
WGS: whole-genome sequencing
