# Supplementary material for: Genomics reveals multiple introductions of the seventh pandemic Vibrio cholerae O1 El Tor lineage into Iran since 1965
Source: Microb Genom. 2025 Oct 30;11(10):001551. doi: 10.1099/mgen.0.001551 (PMC12574951; doi:10.1099/mgen.0.001551)
Supplement: Uncited Supplementary Material 1. [file mgen-11-01551-s001.pdf]

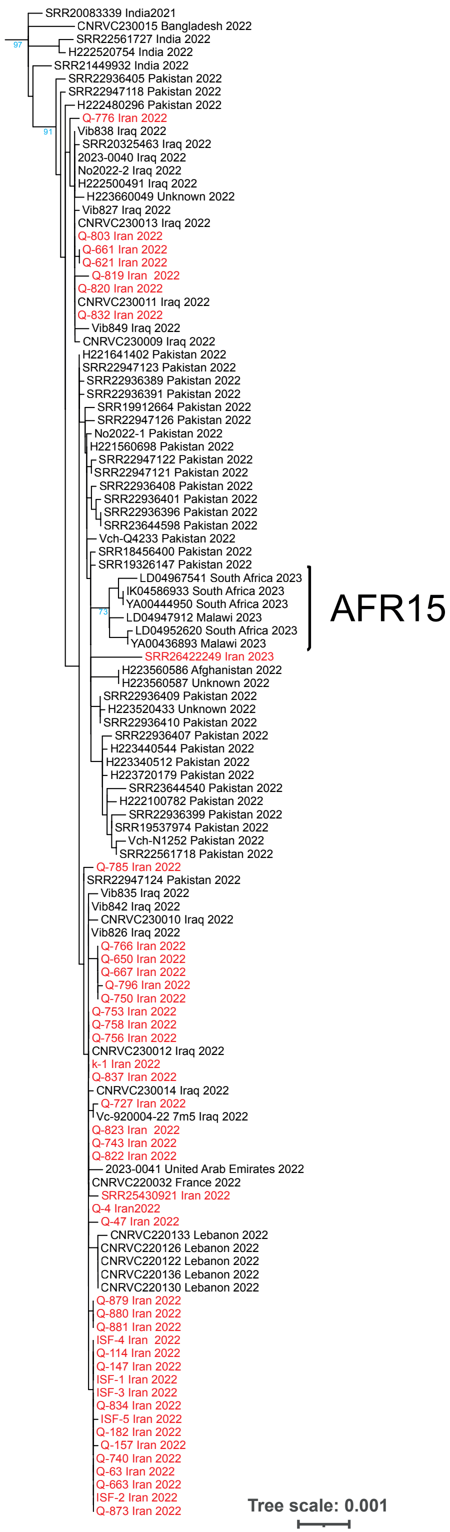
SRR20083339 India2021
CNRVC230015 Bangladesh 2022
97
SRR22561727 India 2022
H222520754 India 2022
SRR21449932 India 2022
SRR22936405 Pakistan 2022
SRR22947118 Pakistan 2022
H222480296 Pakistan 2022
Q-776 Iran 2022
91
Vib838 Iraq 2022
SRR20325463 Iraq 2022
2023-0040 Iraq 2022
No2022-2 Iraq 2022
H222500491 Iraq 2022
H223660049 Unknown 2022
Vib827 Iraq 2022
CNRVC230013 Iraq 2022
Q-803 Iran 2022
Q-661 Iran 2022
Q-621 Iran 2022
Q-819 Iran 2022
Q-820 Iran 2022
CNRVC230011 Iraq 2022
Q-832 Iran 2022
Vib849 Iraq 2022
CNRVC230009 Iraq 2022
H221641402 Pakistan 2022
SRR22947123 Pakistan 2022
SRR22936389 Pakistan 2022
SRR22936391 Pakistan 2022
SRR19912664 Pakistan 2022
SRR22947126 Pakistan 2022
No2022-1 Pakistan 2022
H221560698 Pakistan 2022
SRR22947122 Pakistan 2022
SRR22947121 Pakistan 2022
SRR22936408 Pakistan 2022
SRR22936401 Pakistan 2022
SRR22936396 Pakistan 2022
SRR23644598 Pakistan 2022
Vch-Q4233 Pakistan 2022
SRR18456400 Pakistan 2022
SRR19326147 Pakistan 2022
LD04967541 South Africa 2023
IK04586933 South Africa 2023
YA00444950 South Africa 2023
LD04947912 Malawi 2023
LD04952620 South Africa 2023
YA00436893 Malawi 2023
AFR15
SRR26422249 Iran 2023
H223560586 Afghanistan 2022
H223560587 Unknown 2022
SRR22936409 Pakistan 2022
H223520433 Unknown 2022
SRR22936410 Pakistan 2022
SRR22936407 Pakistan 2022
H223440544 Pakistan 2022
H223340512 Pakistan 2022
H223720179 Pakistan 2022
SRR23644540 Pakistan 2022
H222100782 Pakistan 2022
SRR22936399 Pakistan 2022
SRR19537974 Pakistan 2022
Vch-N1252 Pakistan 2022
SRR22561718 Pakistan 2022
Q-785 Iran 2022
SRR22947124 Pakistan 2022
Vib835 Iraq 2022
Vib842 Iraq 2022
CNRVC230010 Iraq 2022
Vib826 Iraq 2022
Q-766 Iran 2022
Q-650 Iran 2022
Q-667 Iran 2022
Q-796 Iran 2022
Q-750 Iran 2022
Q-753 Iran 2022
Q-758 Iran 2022
Q-756 Iran 2022
CNRVC230012 Iraq 2022
k-1 Iran 2022
Q-837 Iran 2022
CNRVC230014 Iraq 2022
Q-727 Iran 2022
Vc-920004-22 7m5 Iraq 2022
Q-823 Iran 2022
Q-743 Iran 2022
Q-822 Iran 2022
2023-0041 United Arab Emirates 2022
CNRVC220032 France 2022
SRR25430921 Iran 2022
Q-4 Iran2022
Q-47 Iran 2022
CNRVC220133 Lebanon 2022
CNRVC220126 Lebanon 2022
CNRVC220122 Lebanon 2022
CNRVC220136 Lebanon 2022
CNRVC220130 Lebanon 2022
Q-879 Iran 2022
Q-880 Iran 2022
Q-881 Iran 2022
ISF-4 Iran 2022
Q-114 Iran 2022
Q-147 Iran 2022
ISF-1 Iran 2022
ISF-3 Iran 2022
Q-834 Iran 2022
ISF-5 Iran 2022
Q-182 Iran 2022
Q-157 Iran 2022
Q-740 Iran 2022
Q-63 Iran 2022
Q-663 Iran 2022
ISF-2 Iran 2022
Q-873 Iran 2022
Tree scale: 0.001
